# Phospholipids Induce Conformational Changes of SecA to Form Membrane-Specific Domains: AFM Structures and Implication on Protein-Conducting Channels

**DOI:** 10.1371/journal.pone.0072560

**Published:** 2013-08-16

**Authors:** Zhipeng You, Meijiang Liao, Hao Zhang, Hsiuchin Yang, Xijian Pan, John E. Houghton, Sen-fang Sui, Phang C. Tai

**Affiliations:** 1 Department of Biology and Center of Biotechnology and Drug Design, Georgia State University, Atlanta, Georgia, United States of America; 2 School of Life Sciences, Center for Structural Biology, Tsinghua University, Beijing, China; Institut Pasteur Paris, France

## Abstract

SecA, an essential component of the Sec machinery, exists in a soluble and a membrane form in *Escherichia coli*. Previous studies have shown that the soluble SecA transforms into pore structures when it interacts with liposomes, and integrates into membranes containing SecYEG in two forms: SecA_S_ and SecA_M_; the latter exemplified by two tryptic membrane-specific domains, an N-terminal domain (N39) and a middle M48 domain (M48). The formation of these lipid-specific domains was further investigated. The N39 and M48 domains are induced only when SecA interacts with anionic liposomes. Additionally, the N-terminus, not the C-terminus of SecA is required for inducing such conformational changes. Proteolytic treatment and sequence analyses showed that liposome-embedded SecA yields the same M48 and N39 domains as does the membrane-embedded SecA. Studies with chemical extraction and resistance to trypsin have also shown that these proteoliposome-embedded SecA fragments exhibit the same stability and characteristics as their membrane-embedded SecA equivalents. Furthermore, the cloned lipid-specific domains N39 and M48, but not N68 or C34, are able to form partial, but imperfect ring-like structures when they interact with phospholipids. These ring-like structures are characteristic of a SecA pore-structure, suggesting that these domains contribute part of the SecA-dependent protein-conducting channel. We, therefore, propose a model in which SecA alone is capable of forming a lipid-specific, asymmetric dimer that is able to function as a viable protein-conducting channel in the membrane, without any requirement for SecYEG.

## Introduction

The mechanisms of protein secretion in bacteria have been studied extensively in the last few decades. Genetic and biochemical approaches have led to the identification and characterization of a multi-component secretion apparatus, involving SecA, SecYEG, SecB, and SecDFYajC [1]. SecA is an ATPase, and it couples the energy of essential ATP hydrolysis to translocate proteins across the membranes [2–5]. As such, SecA plays a central role in the protein translocation process and interacts with critical components of the translocation machinery, including ATP, proteins, SecB, SecYE, SecG, SecDF•YajC, and anionic phospholipids [6–9]. *In vivo* and *in vitro* studies have shown that SecE- and SecY- deficient membranes are active in protein translocation, indicating that SecYEG is neither the sole-, nor an essential- component of the Sec-dependent translocation machinery for all proteins [1,10–13]. In addition, we have shown that SecA, upon interaction with anionic phospholipids, forms ring-like pore structures [14], which are translocationally active and may form part of the protein-conducting channel itself [14,15]. Recently, we showed that SecA-liposomes alone can promote protein translocation and elicit ion-channel activity [16,17]. SecA most likely functions as a homodimer of 102-kDa subunits [18–20], and exists in soluble and membrane-bound forms within the cells [21]. The cytosolic soluble SecA has two distinct tryptic domains, an N-terminal 68-kDa (N68; residues 1-609) and a C-terminal 34-kDa domain (C34; residues 610-901) [22–26]. The former is an ATPase N-terminal domain that contains two nucleotide-binding regions (NBD1 and NBD2); while the latter appears to function as an ATPase regulator [24]. Proteolytic analyses indicate that SecA undergoes a conformational change upon binding with ATP, precursor proteins, SecYEG, and inverted membrane vesicles [27–31]. It has been reported that N-terminal and C-terminal domains of SecA insert into membranes at SecYEG sites, hydrolyze the bound ATP, and then retract out of the membrane upon release of the translocated protein from SecA. It is through this cycle of insertion and retraction at SecYEG sites that SecA is thought to drive protein translocation [31–33]. Recent studies, however, have found that SecA not only operates as a motor-like component [1], but may also play a structural role in protein translocation [14]. As SecA inserts deeply into membranes many domains of its protein structure, including the C-terminus, are exposed to the periplasmic surface of the *E. coli* inner membranes [34–37]. This deep penetration of SecA into the membrane is promoted by anionic phospholipids [7,38,39].

We have previously found that SecA has two membrane-integral forms [40]; SecA_S_ (a membrane-integral SecA that retains a conformation similar to that of soluble SecA) and SecA_M_ (a membrane-integral SecA with a membrane-induced, protein conformation). Proteolysis of SecA_S_ in the membrane gives rise to an N-terminal 68 kDa fragment and a C-terminal 30-kDa fragment that are apparently similar to the fragments that result from a limited proteolysis of free SecA in solution. Proteolysis of SecA_M_ in the membrane, however, yields two distinctively membrane-specific domain fragments, N39 (residues 1-350) and M48 (residues 361-805), which correspond to the N-terminal and middle portions of the protein. Since formation of these domains is induced by interaction with membranes and is independent of ATP or protein translocation, it has been suggested that these translocation-independent SecA domains may form the constant part of the membrane channel [22,40]. It is, therefore, of great interest to define characteristics of the formation of N39 and M48 domains, especially in light of the recent findings that SecA-alone forms a functional protein-conducting channel in liposomes [16,17], and that SecA functions as a dimer within the membrane [20,41,42,43,44,45,46], most likely an asymmetric dimer [22,42,43].

Here we investigate the formation of the lipid-specific N39 and M48 domains by limited proteolysis in liposomes. We show that liposomes containing anionic lipids are optimal for the formation of these lipid-specific domains. We further show that the N-terminal region of SecA is important for maintaining these domains, not the C-terminus, and that other membrane proteins stabilize their formation. Additionally, atomic force microscopic (AFM) observations reveal that when truncated N39 and M48 protein constructs are exposed to phospholipids they adopt partial ring-structures that are reminiscent of the rings that SecA forms under similar conditions. Based on these and earlier findings, we propose a model for SecA functioning as a protein-conducting channel.

## Materials and Methods

### Bacteria strains

BA13 [19], a sec *A13*(*am*) *supF*(*ts*) mutant and MC4100 were from D. Oliver. *E. coli* RR1/pMAN789-Ns and pMAN789-Cs [26] were from S. Mizushima, *E. coli* PS289 (MC1000, *leu* ^*+*^ *, ara* ^*+*^ *, phoAΔPvuII*, *pcnB80, zadL::Tn10* (Tc^s^ Str^r^)*, secE*Δ19-111*, recA::cat/*pBAD22 *secE* ^*+*^) [47] and its wild-type *E. coli* MC1000 were from C. Murphy and J. Beckwith. The rabbit region-specific SecA antibodies, A_2_ (SecA _211-350_) and A_5_ (SecA _665-820_), were prepared in our laboratory from the plasmid constructs from D. Oliver [22,37].

### Buffers and Media

The following buffers were used where indicated: DTK buffer (1 mM, dithiothreitol, 10 mM Tris-HCl, pH 7.6, 50 mM KCl); DTKM buffer [1 mM dithiothreitol, 10 mM Tris-HCl, pH 7.6, 50 mM KCl,10 mM Mg(OAC)_2_]; DE_20_ (1 mM DTT, 20 mM EDTA); LinA and MinA media were prepared as described [22,40].

### Biochemicals

Gel media for protein purification (S-Sepharose, Q-Sepharose, and Sephacryl S-300) were from GE Pharmacia Biotech Inc. Trypsin, treated with Nα-p-tosyl-L-lysine chloromethyl ketone, and all other chemicals are reagent grade, unless indicated otherwise, obtained from Sigma. [^35^S] protein labeling mix (Expre [^35^S] [^35^S], 1175 Ci/mmol) was from DuPont NEN. The plasmid pET-5a was from Promega. The lipids were from Avanti Polar Lipids, Inc; CL, Cardiolipin (Heart-disodium salt) in chloroform; EM, *E. coli* total lipid extract in chloroform; PE, L-α-Phosphatidylethanolamine (*E. coli*) in chloroform; PG, 1,2-Dioleoyl-sn-Glycero-3-[Phospho-rac-(1-glycerol)] (sodium salt) in chloroform and PC, 1,2-Dioleoyl–sn–Glycero–3-Phosphocholine powder).

### Purification of [^35^S] SecA

[^35^S] SecA was purified from BL21(λDE3)/pT7-*secA* according to procedures described previously [19]. Cells were grown in MinA medium at 37 ^o^C [40]. IPTG (0.5 mM) was used to induce SecA overexpression at O.D._600_ of 0.5. After 10 min, [^35^S] protein labeling mix was added (1 mCi/liter) and the culture was incubated for an additional 2 hrs. The harvested cells were washed once with DTKM buffer, re-suspended in DTKM buffer containing 5 µg/ml DNase and 0.5 mM PMSF, and lysed by passing through a French Press at 15,000 psi. The cell lysates were centrifuged at 8,000 rpm for 15 min. to remove the debris, followed by another centrifugation at 55,000 rpm for 90 min in a Beckman Ti-70 rotor to remove ribosomes and membranes. The supernatant S100 was loaded onto an S-Sepharose column equilibrated with 25 mM phosphate buffer, pH 6.4, and was eluted by a NaCl gradient. [^35^S] SecA was eluted at 0.3 M NaCl. The fractions containing [^35^S] SecA were precipitated with ammonium sulfate. The pellet was then dissolved in 0.15 M ammonium bicarbonate buffer and loaded onto a Sephacryl S-300 column. The final SecA product was virtually pure, as determined by SDS-PAGE and visualized by autoradiography.

### Preparation of inverted membrane vesicles

Membrane vesicles from *E. coli* MC1000, MC4100 and SecA amber mutant, BA13, were prepared by our standard membrane preparation procedures [40,48]. SecE-depleted membrane was prepared from SecE mutant strain PS289 according to procedures previously described [49]. The amount of SecA, SecY, SecE and SecG were determined by immunoblots (The SecE-depleted membranes used in the preparation contained SecE < 1%; SecY < 1%; SecG 1%; SecA 650%, as compared to MC1000 normal membranes).

### Liposomes preparations

1 mg of lipids (in chloroform) were dried in a speed vacuum (Automatic Enviroment SpeedVac system AES1010-120), resuspended in 50 µl of 50 mM Tris-HCl (pH 8.0) buffer, and sonicated (output at 6 with BRANSON Sonifier 450 from VWR Scientific Company) in a water bath at 4 ^o^C for 15 min. PC/PE- was mixed at 1:1; PC/PG, PC/CL PE/CL and PE/PG: were mixed at ratios of 3:1.

### Purification of SecA and truncated SecA proteins

*E. coli* was purified from BL21(λDE3)/pT7-*secA* [31], cultured at 37 ^o^C in LinA medium supplemented with 0.5% glucose, and induced with IPTG (0.5 mM) at O.D._600_ of 0.5. The culture was incubated for additional 2 hrs. Other procedures were the same as that of [^35^S] SecA, as previously described [22,40,41]. N95 (residues 1-831), C34 and C28 (residues 662-901) were similarly purified from soluble fractions using S-Sepharose column and followed by gel-filtration. C95 (residues 64-901) was purified according to Breukink et al [50], and N53 (residues 1-462) and N39 were purified according to Matsuyama et al [26]. The cloned M48*-kDa (residues 344-810) with 6 His-tag in C-terminal was PCR-amplified from pT7SecA with the addition of NdeI and BamHI and ligated into pET-5a. It was then treated with the same enzymes. The PCR primers were 5’-primer: GCT CAT ATG CAG GGC CGT CGC TGG TCC G. 3’-primer: GGA TCC TTA ATG ATG ATG ATG ATG ATG CAT GGA GAA CGA TTC ACG. The cloned M48*-kDa was over-expressed in BL21(λDE3) strain and purified with Ni-NTA agarose (QIAGEN) according to the manufacturer’s instructions.

### Purification of CvaA protein

The gene of *cvaA* [51] was cloned by PCR. His_6_-tag was added to its C-terminus. Primers for *cvaA* gene were 5’ forward (GGGAATTCCATATGTTTCGCCATG ATGCTTTAGAAAAC) and 3’ reverse primer (CCGGAATTCTTAATGATGATGATGATG ATGTCA TTGATCGGTCCTGTTGCACTGTG). The gene of *cvaA* was inserted into the pET-5a vector to yield pET5a/*cvaA*. *E. coli* HMS174 containing pET5a/*cvaA* was grown in LinA medium supplemented with 0.5% glucose and ampicillin (100 µg/ml) at 37 °C. Then 0.5 mM IPTG was added at OD_600_ of 0.6. Cells were harvested after 2 hrs of further incubation. CvaA was overproduced as insoluble inclusion bodies and purified following the manufacturer’s protocols (Xpress^TM^ System Protein Purification provided by Invitrogen Corporation).

### Sequencing of SecA fragments

SecA (10 µg) and N95 (10 µg) were digested with trypsin at 1 µg/ml on ice for 15 min in 100 µl DTK buffer in the presence or absence of lipids (20 µg). The reaction was stopped with 10% TCA. SecA fragments were separated by SDS-PAGE, transferred to PVDF membrane sheets, and visualized by Coomassie Blue staining. Individual bands were then excised from the PVDF membrane sheets and subjected to N-terminal peptide-sequencing analysis by Edman degradation in the Core Facility of Biology Department, Georgia State University. When the excised band contained multiple peptides, all possible amino acids were called and aligned against the known sequences of SecA [52].

### Proteolysis of soluble and lipid-integrated SecA

Trypsin at the indicated amounts was added to DTK buffer containing SecA in the presence or absence of various lipids, (or ATP, or AMP-PNP as indicated) and incubated on ice for 15 min. The reaction was stopped with an equal volume of 20% cold TCA and incubated on ice for 30 min. The precipitates were recovered by centrifugation at 14,000 rpm for 10 min in a table-top centrifuge, washed with 1 ml of cold acetone, and air dried. SecA fragments were then separated by SDS-PAGE, and visualized either by Coomassie Blue staining or by Western blotting with anti-SecA rabbit serum after being transferred to polyvinylidene difluoride (PVDF) membrane (ProBlott; Applied Biosystems).

### Proteolysis patterns of SecA variants

The proteolysis patterns of soluble and lipid-associated N95 and C95 were analyzed as described above Since C95 was prepared in 8 M urea, for comparison, SecA and N95 were also denatured in 8 M urea. All 3 preparations were renatured by dialysis in DTK buffer, then proteolysis was carried out as described above.

### In vitro protein translocation

The translocation of pOmpA into liposomes was conducted as described previously [16]. Unless otherwise indicated, the translocation mixtures in 0.1 ml contained 120 µg of liposomes, 10 µg of C95, or N95, or SecA,150 ng pOmpA and ATP mixtures. The mixtures were incubated at 37 ^o^C; 30 min for pOmpA. The translocation mixtures were treated with proteinase K at 400 µg/ml in ice water for 30 min, and liposomes were collected by centrifugation. Translocated proteins were detected by immunoblots as described previously [16].

### Stabilization of membrane proteins in M48 formation

[^35^S] SecA (1 µg) was incubated with liposomes (20 µg) prepared from *E. coli* lipid mixtures, in the presence of various amounts of proteins including BSA, CvaA, N39, N53, C28 or cytochrome C oxidase (a gift of C.A. Yu), in 100 µl of DTK buffer (pH 8.0) at 37 °C for 15 min. After chilled completely on ice, the reaction mixtures were treated with 20 µl of trypsin (final concentration, 30 µg/ml) on ice for 15 min, and stopped by the addition of cold 10% TCA. SecA fragments were separated by SDS-PAGE. The resulting gels were dried and exposed to Kodak (Rochester, NY.) BioMax MR-1 film for autoradiograms. The lipid-specific [^35^S] M48 band was quantified by Quantity one software (Biorad).

Atomic Force Microscopy: AFM images were obtained with a CP-Autoprobe (Park Scientific, Sunnyvale, CA) using the noncontact mode as previously described [14]. Briefly, SecA and SecA derivatives were mixed by gently vortexing for less than 10 seconds with TKM buffer (10 mM Tris-HCl, pH 8.0, 50 mM KCl and 2 mM MgCl_2_) in the presence or absence of liposomes prepared from *E. coli* total lipid mixtures and incubated in an ice bath for 10 min before being applied onto the freshly calved mica. Samples were incubated at room temperature in a covered dish, rinsed by deionized water for 4 times, and dried in a desiccator until used.

## Results

### Phospholipids Induce SecA to Form Lipid-Specific Domains as Revealed by Limited Proteolysis

We have previously shown that soluble SecA integrates into membrane vesicles upon interaction with membranes [22,40]. Two distinct, membrane-integrated forms of SecA have been revealed by the presence of proteolysis-resistant fragments: one N-terminal 68-kDa fragment that resembles the one produced by limited proteolysis of soluble SecA, and the other M48 that starts at glutamate residue 361. These fragments are specific to SecA in the presence of membranes without protein translocation [22].

**Figure 1:**
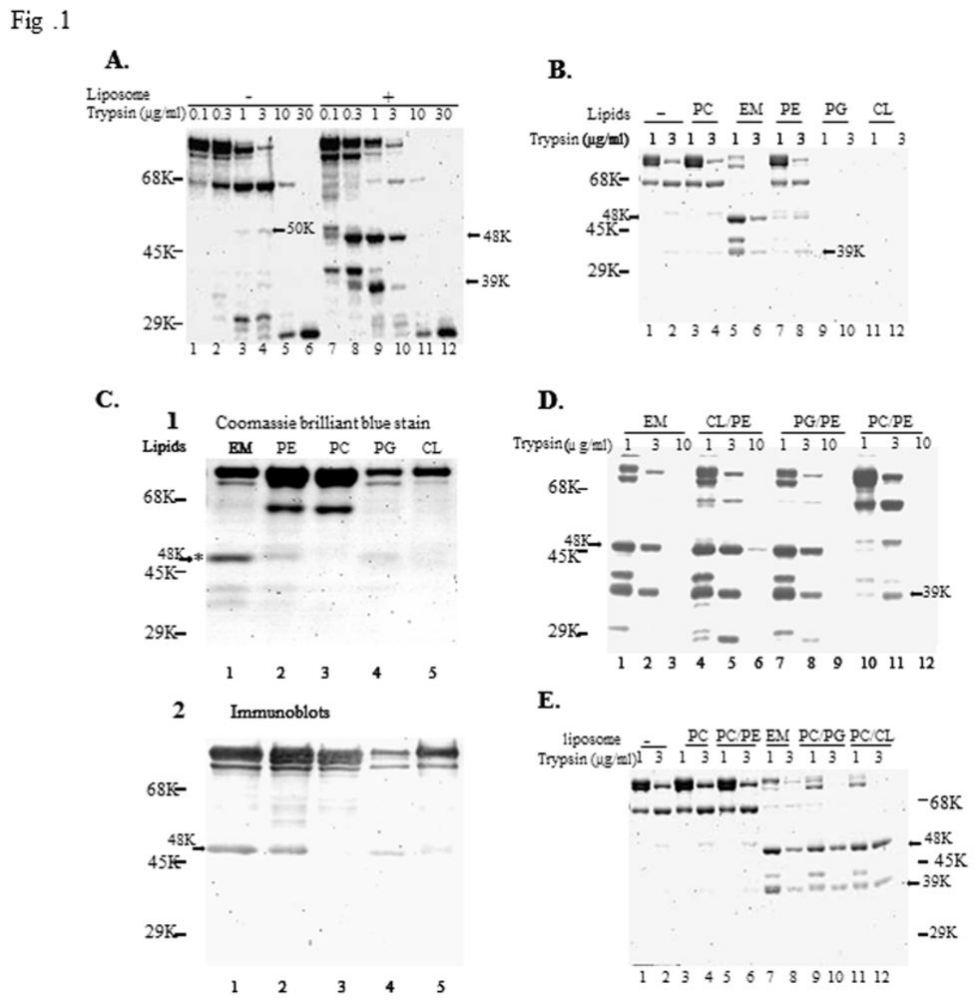
The proteolysis patterns of soluble SecA and various phospholipids-associated SecA. (A) SecA (10 μg) in the absence and presence of liposomes with *E. coli* lipid mixtures (EM) were treated with trypsin at concentrations indicated, as described in the Materials and Methods. The position of the molecular-markers (protein bovine serum albumin (68-kDa), ovalbumin (45-kDa), and carbonic anhydrase (29-kDa)) is shown by bars in the left hand margin. (B) Proteolysis of SecA was carried out (as defined in panel A) in the absence and presence of variously formed liposomes at concentrations of trypsin indicated. (C) Proteolysis of SecA undertaken (as defined in panel B) using 3 µg/ml; with half of the sample subjected to staining with Coomassie blue and the other transferred to PVDF membrane sheets and developed by immunoblotting with a specific antibody against SecA_665-820_ -A_5_. (refs.^6^,^9^). (D) and (E) Proteolysis of SecA was carried out according to a similar protocol used in panel A, except that different mixtures of lipids were used (see text).

To examine the effects of phospholipids on SecA conformation, we analyzed the proteolytic profiles of SecA in the presence and absence of phospholipids. The overall sensitivity of SecA to low concentrations of trypsin differed little in the presence or absence of liposomes prepared from *E. coli* lipids. However, the profiles of proteolytic fragments, as determined by SDS-PAGE, were distinct (Figure 1A): at 1-3 µg/ml of trypsin, soluble SecA gave rise to a major N-terminal 68-kDa band and several smaller fragments, including a 50-kDa fragment (Figure 1A, lanes 3, 4) having an N-terminal sequence of ^9^VFGSRN (Table 1). In contrast, liposomes comprised of phospholipid mixtures induced the formation of two specific domains (Figure 1A, lanes 9, 10); one at M48 starting at ^361^EGVQIQN, and the other at 39-kDa. The domains were mixtures of N-terminal fragments (Table 1). These were similar to the domains of the membrane-specific SecA_M_ form [22,40]. The other 68-kDa and the smaller 27-30-kDa fragments (Figure 1A, lanes 9, 10) were probably integral to the SecA_S_ form, as they were similar to those derived from the cytosolic form of SecA (Figure 1A, lanes 3, 4). The 39-kDa mixtures could be derived from either SecA_M_ or SecA_S_ [22].

**Table 1:** Amino acid sequence analysis of *E. coli* SecA fragments^a^

SecA fragment	SecA+liposome	SecA-liposome	N95+liposome
39 kDa	^1^MLIKLLTK(80%)		^1^MLIKLLtK(44%)
	^9^VFGSRnDR(20%)		^9^VfGSRNDR(39%)
			^14^NDRtLRRM(17%)
48 kDa	^361^EGVQIQNE		
50 kDa		^9^VFGSRNDR	

^a^ *E. coli* SecA (EcSecA) fragments (Fig. 1.A. lanes 3 for 50-kDa, lanes 9 for 39-kDa and 48-kDa; Fig. 2A, lane 2 for 39-kDa with N95) were subjected to peptide-sequencing analysis. Identified SecA sequences were shown in single-letter code. Upper case letters represent amino acids of the analyzed sequences which match the known EcSecA sequence, whereas lower case letters represent unconfirmed residues.

The specificity of phospholipids to the formation of the lipid-specific M48 and N39 domains (as defined by the presence of trypsin-resistant fragments) was determined. The presence of PC had little effect (Figure 1B, lanes 3, 4), and PE alone had only a slight effect (lanes 7, 8), producing a minor M48 band (Figure 1C, lane 2). PE alone did not form liposomes. This suggests that the effect of phospholipids on forming trypsin resistant bands may be subject to binding. The major anionic phospholipids, PG and CL alone not only failed to promote the formation of lipid-specific M48 and N39 domains, but also induced SecA to adopt a conformation that was even more susceptible to trypsin digestion (Figure 1B, lanes 9-12), even though anionic phospholipids are necessary for SecA to function [53]. *E. coli* membrane phospholipids consist of 25-30% anionic lipids (PG and CL) and 70-75% PE [54], and we found that the ratio of negatively charged lipids at 75% with combination of 25% PG or CL yields the similar results as wild-type membranes. Therefore the effects of phospholipid mixtures were determined at PE: PG or PE: CL ratios of 3:1 to mimic the physiological ratios of anionic phospholipids. Both combinations promoted the conformational changes to form the lipid-specific domains similar to the *E. coli* phospholipid mixtures (Figure 1D). While PC could replace PE in these combinations (Figure 1E, lanes 9-12), PC/PE mixtures could not (Figure 1D & 1E).

These data indicate that the formation of phospholipid-bilayer liposomes using anionic phospholipids, PG and CL, in combination with PE or PC (in proportional amounts similar to *E. coli* phospholipid membrane mixtures) promote conformational changes in SecA that induce the formation of the lipid-specific domains of M48 and N39. As previously reported [7,22,39], ATP or AMP-PCP had no significant effect on the formation of these membrane-specific domains (data not shown). All subsequent work was, therefore, carried out using liposomes consisting of *E. coli* phospholipid mixtures (CL: PG: PE ratio about 10:20:70) in the absence of nucleotides.

### The N-Terminal Residues, Not the C-Terminus of SecA Are Required for the Formation of Lipid-Specific Domains, and for Protein Translocation

**Figure 2:**
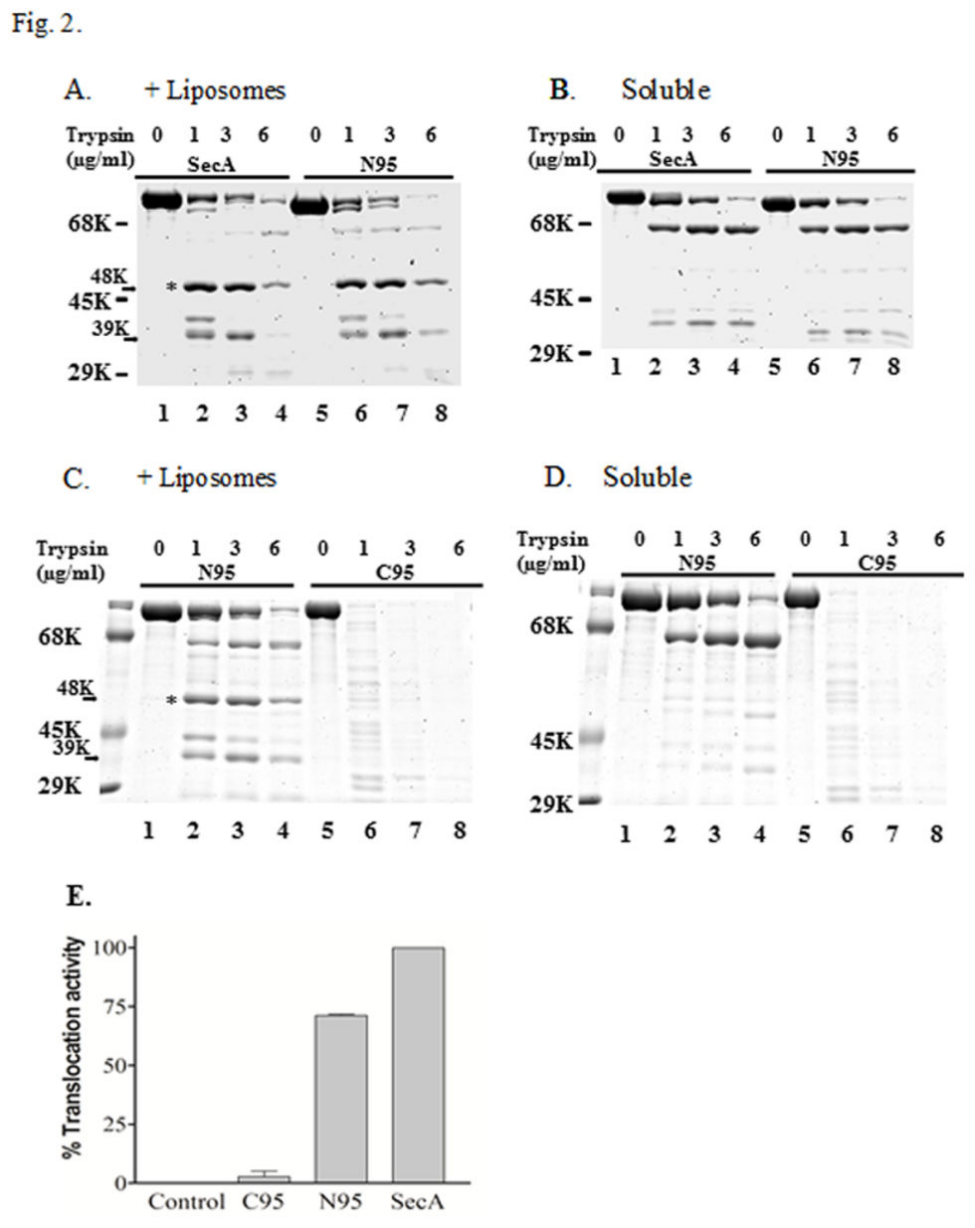
The importance of N-terminal, but not C-terminal, SecA for M48 formation and protein trnslocation. (A) Proteolysis of SecA and the N95 construct was carried out in the presence of EM (as described in the Materials and Methods) with the indicated concentration of trypsin. (B) Proteolysis of soluble SecA and the N95 construct was carried out (as defined in panel A) at the indicated concentration of trypsin (C) Proteolysis of EM-associated N95 construct and C95 were carried out (as defined in panel A) with the indicated concentration of trypsin (D) Proteolysis of soluble N95 and C95 constructs was carried out (as defined in panel A) with the indicated concentration of trypsin. (E) Translocation of pOmpA in the liposomes.

The lipid-specific M48 domain lies in the middle of the 901 residues of SecA -starting at Glu 361 and presumably ending at Arg805 [ [22] and Table 1]. Of the potential membrane-interacting domains of SecA [2,55,56], one domain lies at the N-terminus, while the other domains lie at the C-terminus where they have been shown to interact with SecB and lipids [37,55]. We therefore examined the possible roles of the two extreme termini in the formation of lipid-specific domains. Two SecA deletion variants, C95 and N95 that have the potential to form the M48 fragment (residues 361-805), were purified [26] and examined for their interactions with *E. coli* phospholipid liposomes. N95, which lacks the last 70 residues of the C-terminal lipid-interacting domain, could generate the lipid specific M48 (Figure 2A) and N39 N-terminal mixtures (Table 1), while C95, which lacks the N-terminal 63 residues, could not (Figure 2C). Indeed, the C95 SecA variant showed quite different proteolytic sensitivity and profile from that of either N95 or SecA in the presence or absence of liposomes (Figure 2B–D). These data show that the N-terminal 63 amino acid residues, but not the C-terminal 70 residues, are critical for the formation of SecA domains, including specific interaction with phospholipids. These results correspond well with *in vivo* and *in vitro* translocation activity that N95 is found to possess, but that C95 does not [26]. The N-terminal residues of SecA play a critical role in pre-protein binding [57]. It is, therefore, reasonable to assume that the formation of lipid-specific domains is dependent on the N-terminal residues of SecA. The formation of such lipid-specific domains also corresponded to the ability of N95 to the translocation of pOmpA (Figure 2E) in a SecA-liposome alone translocation system without SecYEG [16].

### Membrane Proteins, Not Necessarily SecYEG, Stabilize the Lipid-Specific Domains of SecA in Liposomes

**Figure 3:**
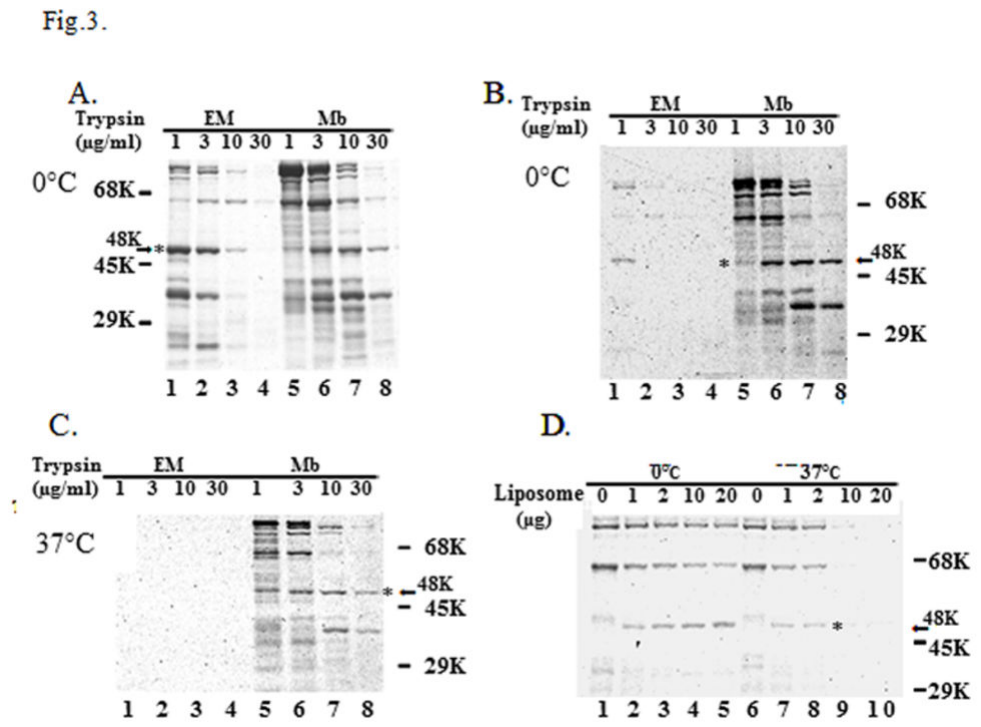
Proteolysis of membrane-integrated SecA and various phospholipid-integrated SecA with trypsin. (A) Liposomes (20 μg) of *E. coli* lipid mixtures and 20 μg BA13 inverted inner membrane (Mb) were mixed with SecA (10 μg) in 100 μl DTK buffer on ice. Trypsin treatment was undertaken at the concentrations indicated. (B) The same reactions were carried out as in panel (A), except that 1 μg of [^35^S]SecA was used. (C) The same reactions were carried out as in panel (A) except that the reactions were incubated at 37 °C for 15 min, chilled on ice prior to the addition of trypsin. (D) 2 μg of [^35^ S]SecA was mixed with different amounts of liposomes (as indicated). Half of the reaction mixtures were incubated at 37 °C for 15 min, while the other half were incubated on ice. All samples were cooled on ice following the reactions. Trypsin (final concentration 1 μg/ml) treatment was used, as described in the Materials and Methods.

Even though *E. coli* phospholipid liposomes induce conformational changes in SecA to form the M48 and N39 domains, the greater sensitivity of these domains to trypsin at concentrations higher than 10 µg/ml (Figures 1 and 2) suggests that other factors, such as interactions of SecA with other membrane proteins, are involved in the resistance of these domains to high concentrations of trypsin (up to 1 mg/ml) when they are embedded in *E. coli* membranes [31,58]. The proteolytic profiles of liposome- and membrane-associated SecA constructs were further examined. SecA (10 µg) was mixed with either liposomes (20 µg) or SecA-depleted BA13 membranes (20 µg lipids) on ice with trypsin for 15 minutes. Though their proteolytic profiles appear to be similar, the lipid-specific M48 and N39 domains of liposome-associated SecA were not very stable when treated with 30 µg/ml of trypsin. This is in contrast to the stable domains within similarly treated membrane-associated SecA (Figure 3A, lanes 4, 8).

To mimic conditions where active protein translocation takes place, and to provide the necessary sensitivity for detection, radioactive-labeled SecA was used (1 µg SecA / 20 µg membrane proteins) to examine the proteolytic profiles of SecA. Under these conditions, the lipid-specific M48 and N39 domains in liposome-associated SecA were more sensitive to trypsin digestion on ice than in membrane-associated SecA; complete digestion was observed at 10 µg/ml of trypsin treatment with the former, but not the latter (Figure 3B-C). The temperature-dependent protease sensitivity of these domains appeared to be related to the SecA-to–liposomes ratio and their interactions at 37 ^o^C (Figure 3D). Therefore, all subsequent trypsin treatments were carried out on ice. These results again indicate that the lipid-specific M48 and N39 domains of SecA are less stable at 37 ^o^C than their membrane-associated counterparts, and that some other membrane proteins may be involved in the stability of these lipid-specific SecA domains.

**Figure 4:**
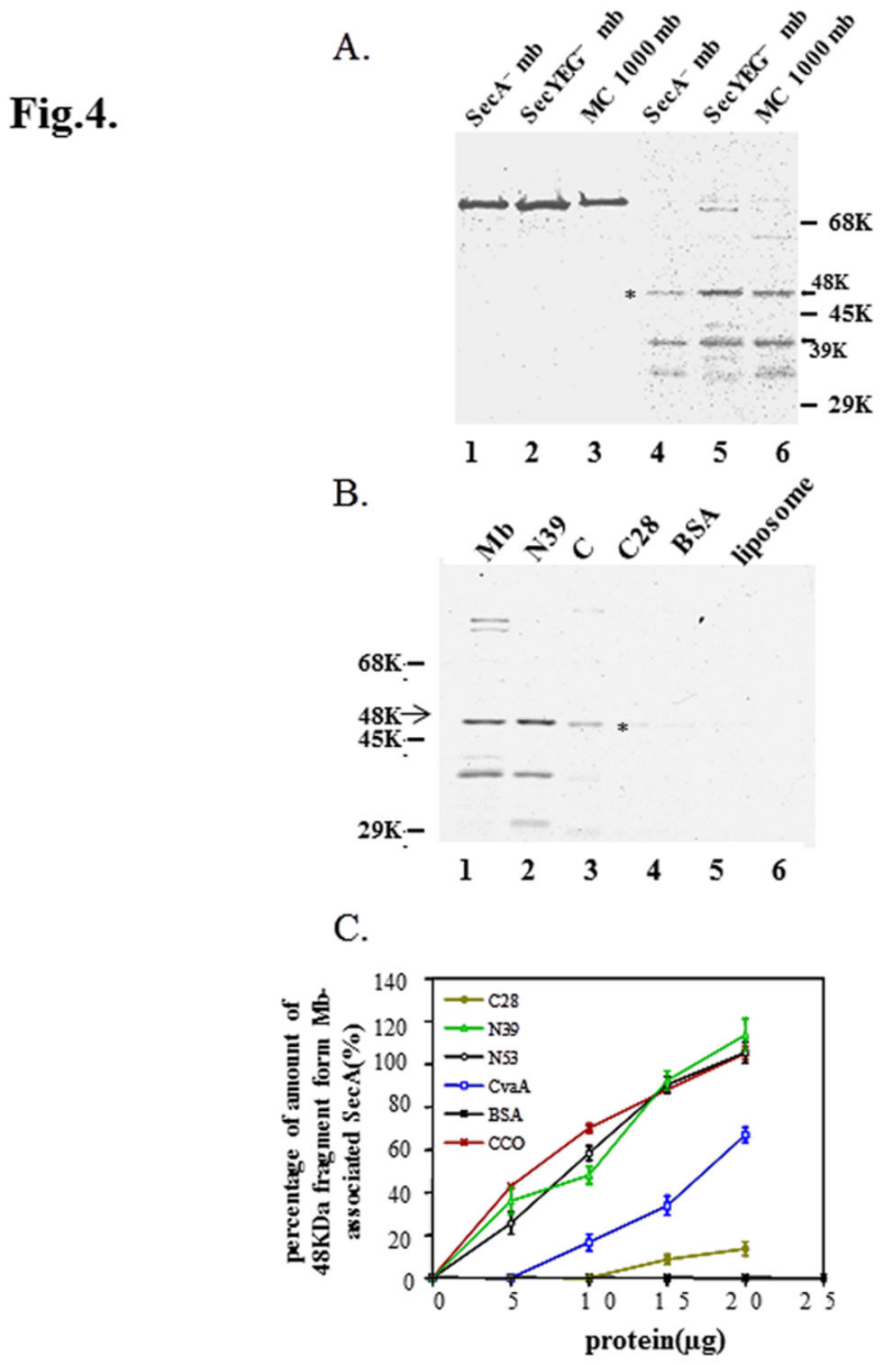
SecYEG are not required for the formation of M48 (A) SecYEG-depleted membrane (20 μg), wild-type MC1000 membrane (20 μg), or SecA-depleted BA13 membrane (20 μg) was mixed with [^35^S]SecA (1 μg) in 100 μl DTK buffer, respectively. After pre-incubation at 37 °C for 15 min, the samples were cooled on ice and incubated for 15 mins with (lanes 4-6) or without 30 µg/ml trypsin (lanes 1-3). (B) The reactions were carried out, as in panel A, in the presence of 20 μg of membranes, N39, C28, BSA or Cytochrome C (lane 3) or liposomes alone (lane 6). (C) The reactions were carried out (as in panel B) in the presence of various amounts of N39, N53, C28, CvaA, BSA, and CCO (Cytochrome C Oxidase). The amount of M48 domain fragment in each liposomal fraction was quantitated after gel electrophoresis relative to the amount of the M48 domain obtained from mb-associated SecA (which was taken as 100%). The amount of M48 domain from CCO was calculated from immunoblots while other amounts were calculated from autoradiograms. The data were from 2-4 sets of independent experiments, and presented as mean + SE.

SecYEG complexes have been shown to bind SecA with high-affinity [59,60,61,62]. Therefore, SecYEG complexes are logical candidates to stabilize the SecA domains in the membrane. To determine whether SecYEG is essentially required for the stability of SecA lipid-specific domains, we employed SecE-depleted membranes [11] that contained no detectable SecE or SecY and little SecG (< 1% of wild-type MC1000 membranes) as determined immunologically. Surprisingly, the SecYEG-depleted membranes stabilized the M48 and N39 domains (1 µg of radioactive SecA) as well as wild-type membranes or SecA-depleted BA13 membranes (Figure 4A, lanes 4-6). In the SecYEG-depleted membranes, SecA was overproduced approximately 5-6 fold [11]. As a result, the amounts of SecA and SecYEG in the membrane are not directly related to the stability of lipid-specific domains.

The major difference between liposomes and membranes with the same amount of phospholipids is the presence of equal amounts of proteins in the membrane. We therefore examined whether the addition of proteins to liposomes mimic the enhancement in the stability of SecA domains in the membrane. The addition of soluble bovine serum albumin (Figure 4B and C) or cytochrome C had no stabilizing effect (Figure 4B, lane 3), even though the latter binds loosely to liposomes (at 20 µg). Several fragments of SecA [26] including the N39, N53, M48* as well as C28 were cloned, purified and tested. The purified N-terminal N39 and N53 fragments integrated into the liposomes and stabilized the formation of the radio-labeled tryptic M48 and N39 domains to the same extent as membranes, while C-terminal C28 had little effect (Figure 4B and C). Such stabilizing effects were dependent on the protein concentration for the interaction of liposomes and SecA (Figure 4C). To examine whether the stabilizing factor is restricted to N-terminal SecA fragments, an unrelated purified membrane protein, CvaA, which is a component of ABC transporter for colicin V [63], was used (Figure 4C). Addition of a membrane protein CvaA did indeed stabilize the lipid-specific M48 (Figure 4C) and N39 domains (data not shown) in a concentration-dependent manner, though it was not as effective as the addition of N39 or N53 SecA fragments (Figure 4C). In addition, the presence of another unrelated membrane protein complex, cytochrome C oxidase complex [64] also showed effective protection of stability (Figure 4C).

These results indicate that SecYEG complexes are not essential for stabilizing trypsin-resistant SecA domains in membranes as N-terminal domains of SecA are equally as effective, and even unrelated membrane proteins can also stabilize these lipid-specific domains, albeit with varying degrees of efficiency.

### Properties of Phospholipid-Specific Domains

**Figure 5:**
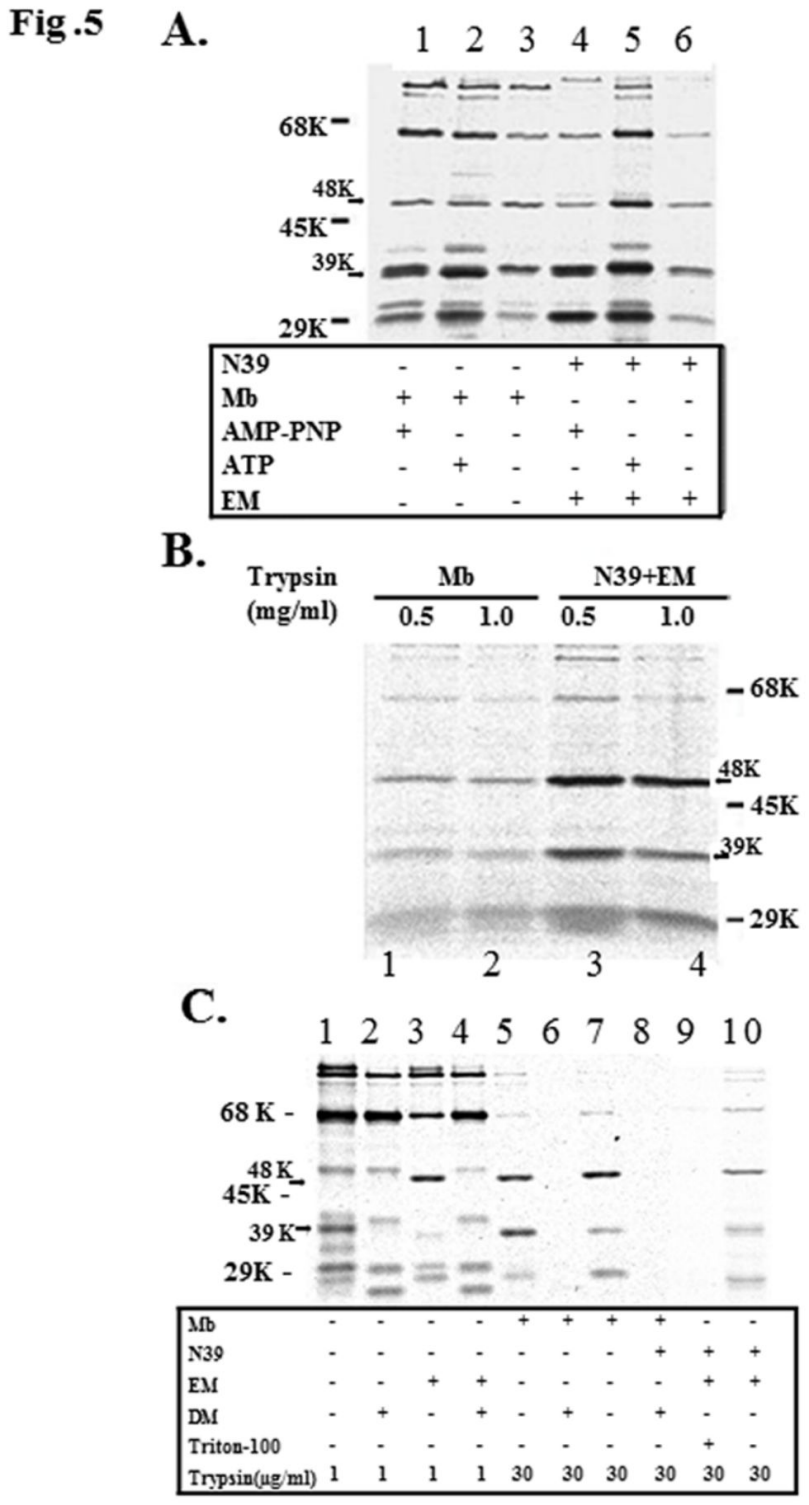
Characterization of the protease-resistant domains from membrane or EM-associated SecA. (A) The effect of nucelotide binding on the stability of the M48 domain was determined using similar proteolysis reaction conditions to those undertaken in Fig. 4, except in the presence of 2 mM ATP or AMP-PNP and 4 mM Mg(OAc)_2_. (B) The stabilization effect of membranes and N39 with EM on the M48 domain was assayed using proteolytic reactions to those carried out in panel A at the trypsin concentrations indicated. (C) The effect that integrity of the membrane has upon stability of the M48 domain was ascertained by undertaking proteolysis at different concentration of trypsin in the presence of Triton-X100 (1%) or malto-dodecylmaltoside (DM, 2%) as indicated. Reaction conditions used were similar to those defined in panel A.

To determine whether the domains induced by proteoliposomes behave differently from those induced by membranes, we examined the effects of ATP and its nonhydrolyzable analog AMP-PCP on the protein’s resistance to high concentration of trypsin in both proteoliposomes and membranes. The presence of either nucleotide had little effect on the formation of M48 and N39 domains (Figure 5A), indicating that the Walker binding sites are not critical for the stability for these lipid-specific domains. These domains were similarly resistant to trypsin treatment even up to concentrations as high as 1 mg/ml (Figure 5B), as found previously [22]. Moreover, the use of heparin to disrupt the ionic interaction of SecA with phospholipids had no effect [22], and Na _2_CO_3_ extraction after trypsin treatment also proved to be similar for the domains in proteoliposomes and in membranes (Table 2). Thus, the N68 and N39 domains could not be extracted by Na _2_CO_3_, indicating the integral nature of their association with liposomes and membranes (even though N68 is soluble without lipids) while the M48 domain was seen to be partially extracted (about 50%), indicating that it is in a more aqueous environment; as reported earlier under conditions of protein translocation [22].

**Table 2:** Nature of liposome-specific domains and membrane-specific domain of SecA^**a**^

	**Membrane (%)**			**Liposomes (%)**			**Liposomes+N39 (%)**		
	TK	Na_2_CO_3_	Heparin	TK	Na_2_CO_3_	Heparin	TK	Na_2_CO_3_	Heparin
SecA	100	123±7.2	68±12	100	130±9.5	81±33	100	115±12	95±34
68kDa	100	113±10	92±23	100	128±7.8	88±31	100	110±25	75±23
48kDa	100	36±4	102±19	100	42±8.3	100±33	100	50±20	106±34
39kDa	100	101±13	100±28	100	130±25	80±36	100	100±17	68±9.3

^a^Reaction mixtures with 1 µg [^35^S]SecA were incubated and treated as in Fig. 5.C. The membranes, liposomes and liposomes with N39 (20 µg/ml) and were recovered by centrifugation and re-suspended in 100 μl of TK buffer, 10 mg/ml heparin or 0.1 M Na_2_CO_3_ (pH 11) respectively. After incubation on ice for 30 min, the liposomes and membranes were recovered by centrifugation and were analyzed by SDS-PAGE and autoradiography. The quantities of remaining SecA fragments in liposomes and membranes were determined by Quantity One software, using these in TK buffer as 100%. All experiments were carried out for 3-5 times.

To determine whether the formation of these domains and their resistance to trypsin were due to the intrinsic resistance of these domains or due to some protection conferred from being formed in the presence of proteoliposomes or membranes, detergents were added after the formation of SecA domains. The non-ionic detergents, Triton X-100 and malto-dodecylmaltoside, which have previously been shown to maintain SecYEG in soluble form and to allow the formation of an inserted 30-kDa SecA domain [33], were used. Neither conferred the subsequent protection to trypsin digestion, indicating that the M48 and N39 domains are specifically induced and maintained only in the presence of phospholipids (Figure 5C).

### Structures of Lipid-Specific Domains as Observed by Atomic Force Microscopy (AFM)

**Figure 6:**
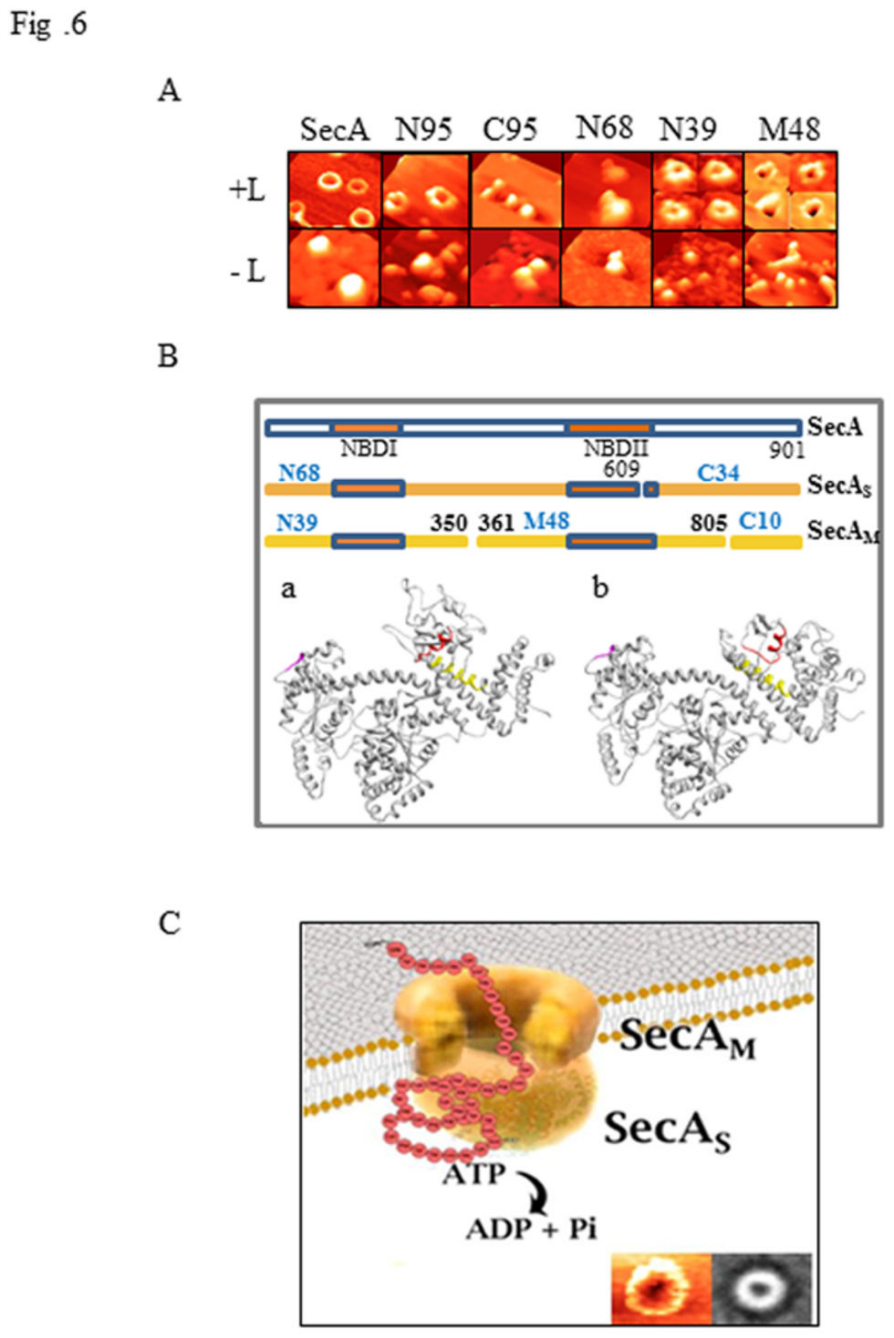
AFM images of SecA and its domains suggesting a model of a SecA channel for protein secretion. (A) Structural variations adopted by purified SecA and a series of its truncated domains were determined by incubating them in the presence and absence of lipid bilayers (+/- L) prepared from *E. coli* extracted lipid mixtures. Sheets of mica were freshly cleaved and samples were prepared as previously described [14]. The upper rights images are a series of zoomed-in images of N39 and M48 incubated in the presence of liposomes and, are not shown to scale. The estimated sizes for SecA, N39 and M48 are 10.3 ± 1.0 nm, 7.1 ± 0.4 nm and 6.2 ± 0.4 nm, respectively, with n>15. (B) Potential trypsin cleavage sites of N68, and N39 and M48, in SecA are shown along with the location of the two nucleotide binding sites, each with its own attendant Walker A and B motifs (orange bars). The location of each of the potential cleavage sites in the crystal lattice structure of SecA in either the open-state (a) or in the closed-state (b) are also shown. The potential cleavage-site 1 (609-613) is colored in magenta, while the cleavage-site forming the N39 region is colored in red and the cleavage-site separating M48 from C10 is colored in yellow. (C) A model as to how the asymmetric dimer of SecA forms a functional channel that allows protein precursors to be translocated across the cytoplasmic membrane and into the periplasm. The two distinct forms of integral SecA (SecA_M_ and SecA_S_) together form the structural channel that can be observed for SecA alone using AFM and TEM (lower right frames). SecA_M_ consists of N39, M48 and C10, and SecA_S_, consists of N68 with ATPase activity and C34, and appears to adopt the same conformation as SecA in its fully soluble form.

We have previously demonstrated the ability of SecA to form ring-like pore structures upon its interaction with phospholipids using both electron microscopy and atomic force microscopy [14]. Here we used AFM as the tool of choice to probe overt conformational changes of the SecA domains that could be induced by phospholipids. In agreement with limited proteolysis results, N95 but not C95 (lacking the first 63 N-terminal residues) formed a ring-like structure similar to those formed by wild-type SecA in the presence of phospholipids (Figure 6A), and protein translocation with liposomes (Figure 2E). These results further support the conclusion that the N- terminal, but not the C-terminal, of SecA is required for the formation of lipid-specific domains. We next examined the structure of various SecA domains in lipids. We cloned, overexpressed, and purified the lipid-specific domains of N39 and M48*, as well as the soluble domain of N68 [24]. The lipid-specific domains of N39 and M48* formed imperfect partial ring structures, which appeared to be smaller than those of the intact SecA, while the soluble N68 domain [24] (Figure 6A) and C34 failed to form any ring structures at all (not shown). All ring structures that were observed were only formed in the presence of phospholipid liposomes with no detergents and did not form in the absence of lipids. The N39 ring structure differed from that formed by the M48* domain in that the former appeared to be more anchored on the surface of the lipid-bilayers with an outward amplitude, whereas the partial M48 ring structures showed a much more deeply indented cavity with an inward amplitude at the center of the ring-structure (Figure 6B). The partial pore structures of the N39 and M48 were smaller than SecA: using gold particles as the standard with AFM, the estimated sizes for SecA, N39 and M48 are 10.3 ± 1.0 nm, 7.1 ± 0.4 nm, 6.2 ± 0.4 nm, respectively. In addition, sequence analysis showed that N39 possesses more hydrophobic area than M48 does. These data are also consistent with the observations that N39 was resistant to Na _2_CO_3_ treatment, while M48 was removed 50% by the same treatment (Table 2). These data suggest that the lipid-specific domains of N39 and M48* do form part of the characteristic ring-like pore structures of SecA following its interaction with phospholipids, and further suggest that these lipid-specific domains are important components of SecA function in the membrane. It is interesting to note that N39 forms partial ring structures while the larger N68 did not, indicating the dynamic nature of the formation and maintenance of these domain structures.

### A Model of SecA as Protein-Conducting Channel in the Membrane

We have earlier found that SecA forms ring-like structure upon interaction with *E. coli* phospholipids [14] and recently we showed that SecA alone can promote protein translocation in liposomes and elicit ion-channel activity [16]. In addition we have also shown that dimeric SecA couples the preprotein translocation in an asymmetric manner [42,43]. This study has demonstrated that SecA, in the presence of liposomes, forms the lipid-specific domains of N39 and M48, with characteristics similar to the membrane-specific domains, in addition to the N68 and C34 domains present in the soluble SecA and membrane SecA_S_ forms [22]. From X-ray structures of soluble *E. coli* SecA [46], the most likely tryptic cleavage site in SecA_S_ that gives rise to N68 and C34 is 608-609RK, which is in a loop at the surface of the protein and probably lies within the Walker B motif of the NBDII region (Figure 6B). As a result of the drastic conformational change with lipids, the cleavage sites for SecA_M_ that give rise to N39 and M48 are probably at 348R in the PBD domain, and 805R in the an α-helix in IRA1 domain (Figure 6B). To account for these observations, we propose a model (Figure 6C) in which the SecA dimer alone functions as the protein conducting-channel in promoting protein translocation and ion channel activity [14,16,17]. In this model SecA adopts an asymmetric dimer configuration in the membrane [42,43], in which the membrane bound subunit SecA_M_ is specific for membrane lipids and plays the structural role of a channel, while the other SecA subunit, SecA_S_, has essentially the same conformation as it does in its soluble form and functions as the ATPase motor. SecA_S_ has both the ATPase domain of N68 and the regulatory C34 domain, while SecA_M_ is structurally composed of the N39 domain forming the surface part of the ring-pore structure and the M48 domain forming the more aqueous part of the channel (Figure 6C), as well as a small C10 domain that is probably exposed to the periplasmic side [35,37]. In their lipid-dependent structural configuration, neither N39 nor M48 exhibit ATPase activity (data not shown), even though each possesses an adequate nucleotide binding domain (NBDI and NBDII, respectively) with each domain encompassing an intact Walker A and B sites (Figure 6B). In this model for SecA-dependent protein translocation, SecA_S_ alone would provide the ATPase activity that is capable of propelling the protein precursor(s) through the SecA_M_ channel. While the proposed model depicted in Figure 6C indicates a clear distinction between the two asymmetric units of the liposome-induced SecA dimer, it is not entirely inconsistent with the possibility of each subunit “flip-flopping” between each other conformation as the protein that is being translocated is propelled into the periplasmic space.

## Discussion

SecA is present in bacterial cells as both a soluble and a membrane form [26,65,66], and has been suggested to cycle on and off the membrane during protein translocation. A number of studies have shown, however, that SecA inserts deeply into liposomes and, in so doing, undergoes a series of conformational changes [7,32,38,67]. In addition, an earlier study from our laboratory also showed that a significant fraction of SecA remains on the membrane and does not cycle on and off during protein translocation [40]. Consequently, it has been suggested that SecA interacts with membranes, resulting in series of conformational changes that allow it to form part of a protein-conducting channel [22,40]. The specific domains that characterize the membrane bound conformation of SecA are the proteolytic N39 and M48 fragments [22]. This study shows that these membrane-specific domains can be induced by liposomes alone.

Formation of the lipid-specific M48 domain (and the corresponding N39 domain) depends on the species and the combination of the phopholipids used. It has been well established that anionic lipids such as PG and CL are required for SecA to function *in vitro* and *in vivo* [3,12,14,59,66,68,69], even though overall there are 26 more negatively charged Glu and Asp residues in the *E. coli* SecA, for example, than there are positively charged Arg and Lys residues [68]. In trying to determine critical characteristics of phospholipids that would facilitate formation of the M48 domain in SecA a variety of phospholipid mixtures were tested. The combinations of PG/PE or CL/PE in 1:3 mixtures were as effective as the liposomes made of extracted *E. coli* lipid mixtures in forming the lipid-specific M48 domain -as defined by limited trypsin proteolysis (Figure 1D). Similarly, liposomes made of PG/PC or CL/PC were also effective (Figure 1E). However, while PC had no discernible effect upon the proteolytic integrity of SecA, the presence of PG or CL phospholipids rendered SecA more susceptible to trypsin proteolysis (Figure 1B). Interestingly, even though PE is incapable of forming liposomes by itself, this positively charged phospholipid was able to induce formation of the lipid-specific M48 domain; albeit less effectively than in combination with either PG or CL. Intriguingly, PE/PC mixtures were unable to induce formation of the M48 domain. These results indicate and that there is a delicate balance in the interactions between these phospholipids and SecA and that SecA is highly sensitive to the composition of liposomes that is able to promote the necessary structural changes for SecA to adopt a lipid-specific SecA_M_ ring-like configuration.

Limited proteolysis and sequence analysis have shown that liposome-embedded SecA gives rise to the formation of the same major M48 and N39 fragments as membrane-embedded SecA [ [22] and Table 1]. Despite this similarity, the liposome-embedded SecA is found to be more sensitive to proteolysis at 37 ^°^C and to lower concentrations of trypsin than membrane-embedded SecA (Figure 3C). While these results are not too surprising, they do suggest that some other membrane proteins would presumably contribute to the stability of the lipid-specific SecA domain under physiological growth conditions. In this regard, N-terminal SecA fragments, as well as unrelated membrane proteins CvaA or cytochrome C oxidase (but not other soluble proteins), were found to stabilize the formation of lipid-specific SecA domains in liposomes as effectively as membrane (Figure 4C). This is particularly noteworthy in that, while SecYEG complexes have been reported to bind SecA with a high affinity [59,60,61,62], SecYEG complexes are not essential for the higher stability of SecA in the membrane, since SecYEG-depleted membranes (with concentrations of SecYEG less than 1%) yield the same lipid-specific SecA proteolytic fragments as those of wild-type membranes (Figure 4A). The requirement of some additional membrane proteins to stabilize SecA conformation underscores the importance of a more rigid state of phospholipids at high temperatures for SecA to function in the membrane. Thus, although anionic liposomes are sufficient to induce the formation of lipid-specific domains in SecA other membrane proteins are required to maintain such conformations under physiological conditions. In this regard, the formation of SecA ring-structures were observed with phospholipids at lower temperatures, albeit less stable at higher temperatures [70]. This requirement for membrane proteins emphasizes the fluidity of phospholipids in the absence of membrane proteins. These proteins may have two functions: 1) SecYEG and SecA N-terminal domains directly interact with SecA to stabilize its conformation; 2) unrelated membrane proteins insert into lipids and stabilize the neighboring liposome structure, which in turn stabilizes the inserted SecA conformations to produce the M48 lipid-specific domain.

The middle M48 domain can be formed by tryptic treatment theoretically from either N95 or C95 that is missing 60-70 aa residues at either end, yet deletion of the N-terminal 60 aa (C95) renders this truncated SecA incapable of forming the M48 proteolytic domain (Figure 2C) and protein translocation (Figure 2E), and unable to complement the temperature-sensitive SecA amber mutation BL21.19. In contrast, deletion of C-terminal 60 aa (N95) of SecA has no effect on the formation of the M48 proteolytic fragment (Figure 2C) and is capable of promoting protein translocation in liposomes (Figure 2E). The additional observation that the N39 fragment is more resistant to chemical extraction when it is embedded in the membrane [22], or in the liposomes (Table 2), is also consistent with the importance of the N-terminus forming the lipid-specific domain structures, especially when, despite the overall abundance of negatively charged amino acids in SecA [68], there are seven more positively charged amino acids in the first 25 N-terminal amino acids than there are negatively charged residues. These results suggest that the N-terminal region of SecA is important in forming the lipid-specific domains and protein translocation (potentially through interactions with the more anionic phospholipids), which is in agreement with previous assertions that the N-terminus of SecA is responsible for high affinity binding [1,11] while the C-terminus demonstrates a low affinity for lipids [4].

Previous studies have shown that SecA forms ring-like pore structures upon interaction with anionic phospholipids, as observed by electron microscopy and Atomic Force microscopy [14]. Such characteristic structures of SecA appear to be dimers [14] that are entirely functional in their interactions with SecB [15], and may form the core of bacterial protein-conducting channels in the membrane [14,48]. The Atomic Force Microscopy observations here show that the lipid-specific domains, N39 and M48, form parts of the characteristic SecA ring-like pore structures: the M48 domain seemingly embedded more deeply into the lipid layers forming a deep pore, while the N39 domain appears to form a more superficial ring structure that is mostly anchored to the surface of the liposome. Since these unique structures were not observed in the absence of phospholipids, and were also not observed in the N68 nor C95 constructs, they would presumably represent parts of the SecA ring-like pore structure that forms the SecA-dependent protein-conducting channel, as proposed in the model of SecA (Figure 6C). It is also worth noting that the N39 domain behaves more as an integral membrane protein than does the M48 domain, which appears to be present in a more aqueous environment, as detailed by its response to chemical extraction [ [22] and Table 2].

The combined chemical properties and physical characteristics of the lipid-specific domains within SecA suggests how this protein readily adapts to the membrane environment; potentially forming a low-affinity channel for bacterial protein translocation in the absence of any high-affinity binding sites (that may ultimately be formed through its interactions with SecYEG [16,17]). The integration of SecA into membranes to form these lipid-specific domains with itself (absent any other protein interactions), represents a fundamentally important development in any understanding of how SecA functions in the membrane. The possible structural roles of the N39 and M48 domains of SecA in supplying critical parts of the protein-conducting channel provide a reasonable explanation to a question that has been elusive until now, as to how SecYEG-deficient membranes are still capable of translocating certain protein precursors [10,11,12,13,71,72]. Blobel’s lab as early as in 1990 (71) showed that SecY/PrlA is not essential for translocation of all precursor proteins. It should be pointed out that SecYEG is not functional without SecA, while SecA can function without SecYEG, albeit less efficiently [16,17]. The model that we propose for SecA to function as a protein-conducting channel in its own right (Figure 6), even in the absence of SecYEG, is strongly indicated by a summation of all the available evidence, and provides a plausible and testable understanding as to how Sec-mediated protein translocation across the cytoplasmic membrane of *E. coli* may occur.
